# Zoonotic Agents in Farmed Fish: A Systematic Review from the Interdisciplinary Perspective of the One Health Concept

**DOI:** 10.3390/vetsci12050437

**Published:** 2025-05-02

**Authors:** Juliana Rosa Carrijo Mauad, Marcelo Corrêa da Silva, Carolina Marques Costa Araújo, Rosilda Mara Mussury Franco Silva, Silvia Morales de Queiroz Caleman, Márcia Regina Russo

**Affiliations:** 1 Postgraduate Program in Agribusiness, Federal University of Grande Dourados, Dourados 79804-970, MS, Brazil; julianacarrijo@ufgd.edu.br; 2 Center for Rural Development, Sustainable Solutions Network, Dourados 79849-899, MS, Brazil; marcelo-correadasilva@hotmail.com; 3 Postgraduate Program in Entomology and Biodiversity Conservation, Federal University of Grande Dourados, Dourados 79804-970, MS, Brazil; maramussury@ufgd.edu.br; 4 School of Business Administration, Federal University of Mato Grosso do Sul, Campo Grande 79070-900, MS, Brazil; silvia.caleman@ufms.br; 5 Postgraduate Program in Biodiversity and Environment, Federal University of Grande Dourados, Dourados 79804-970, MS, Brazil; marciarusso@ufgd.edu.br

**Keywords:** aquaculture, bacterial zoonoses, interdisciplinary, one health, parasitic zoonoses

## Abstract

This systematic review aims to synthesize evidence on zoonotic agents in farmed fish from the interdisciplinary One Health perspective. The review followed a protocol, following the PICOS strategy, where P represents population (farmed fish), I represents intervention (fish diseases), C represents comparison (healthy fish), O represents outcome (One Health) and S represents the type of study (experimental studies). The searches were conducted in the SCOPUS, Science Direct and PubMed Central databases. Of the 400 articles identified, 23 met the inclusion criteria due to their methodological robustness. These articles focused predominantly on parasitic (60.9%) and bacterial (39.1%) zoonotic agents. The One Health approaches discussed throughout the articles included host-parasite interactions (35%), antimicrobial resistance (22%), infections and food safety (18%), nutrition and immune responses (17%), stress and immune responses (4%) and anthelmintic testing (4%). The findings indicate that high-level scientific production is often confined to specific fields, such as veterinary medicine, biological sciences and animal science/fishery resources. There is a lack of broad interdisciplinary collaboration, limiting the integration of diverse fields to improve scientific production.

## Simple Summary

This systematic review synthesizes evidence on zoonotic agents in farmed fish from the interdisciplinary perspective of One Health. Using the PICOS strategy, 400 articles were analyzed from the SCOPUS, Science Direct and PubMed Central databases, 23 of which met the inclusion criteria. The studies focused mainly on parasitic (60.9%) and bacterial (39.1%) zoonotic agents. The One Health approaches addressed included host–parasite interactions (35%), antimicrobial resistance (22%), infections and food safety (18%), nutrition and immune responses (17%), stress and immune responses (4%) and anthelmintic testing (4%). The results indicate that high-level scientific production is mostly restricted to specific areas, such as veterinary medicine, biological sciences and fishery resources, highlighting the lack of broader interdisciplinary collaboration, which limits the integration of different fields to improve scientific production.

## 1. Introduction

Aquaculture production or activities are increasingly contributing to the ecosystem, food security and nutrition of the population [1]. At a global level, exponential population growth, a reduction in extractive fishing and an increase in fish consumption by humans have imposed pressure on the fish sector to intensify its operations or production processes [2,3]. The process of intensification can be associated with intensive and semi-intensive production practices and greater concentrations of animals in smaller aquatic spaces, which are often more stressful or may challenge the immune system and cause an increase in infections [1,4,5].

Infections or diseases can be transmitted from animals to humans or from humans to animals, resulting in their classification as zoonotic diseases [6]. Zoonotic agents include a wide range of bacteria [7,8], parasites [9,10], viruses [11], fungi and protozoa [12].

In fish, most zoonotic diseases or infections are associated with bacteria [12]. One of the bacterial pathogens widely recognized for its zoonotic potential is *Mycobacterium marinum*, which is known to cause mycobacteriosis in both fish and humans. Other species, such as *Mycobacterium fortuitum* and *Mycobacterium chelonae*, are also implicated in similar zoonotic infections, emphasizing the public health risk associated with these bacteria [13,14]. Another notable pathogen is *Edwardsiella tarda*, which is associated with systemic infections in fish and can lead to zoonotic infections in humans [15]. *Vibrio* species, particularly *Vibrio parahaemolyticus* and *Vibrio vulnificus*, are also recognized as important zoonotic pathogens [16].

Studies on *Aeromonas* spp. indicate that these bacteria are emerging pathogens, often associated with gastrointestinal infections, skin and soft tissue infections and even septicemia in immunocompromised individuals [17,18,19,20]. The presence of *Aeromonas* in aquatic environments and in animals such as fish and rodents suggests that these organisms can act as reservoirs of zoonotic pathogens, facilitating transmission to humans [21,22]. In addition, the antibiotic resistance observed in *Aeromonas* is alarming, as it can lead to the spread of resistance genes between different species, increasing the risk to public health [23,24].

*Klebsiella* spp., *Yersinia* spp. [25] and *Vagococcus* spp. [26], although less discussed in zoonotic contexts, are also of growing concern as they cause infections in humans and animals, in addition to their ability to acquire resistance to multiple drugs, raising concerns about food safety and public health [26].

Similar to bacteria, zoonotic parasites are also a public health problem [27,28] and can be classified into the following three main groups: trematodes, nematodes and cestodes [29]. In most cases, the parasites use fish as intermediate hosts and impose energy demands on them that influence their morphology, fecundity, reproduction, behavior and survival [30].

Trematodes, especially from the *Opisthorchiidae* and *Heterophyidae* families, are known to cause infections in humans. Species such as *Opisthorchis viverrini* and *Haplorchis taichui* are notable examples that can be acquired by eating raw or undercooked fish [31,32,33]. In addition, the *Heterophyidae* family includes several species that are responsible for intestinal infections in humans, with more than 30 known species that can parasitize humans [34]. Nematodes, particularly those from the *Anisakidae* and *Gnathostomatidae* families, also have significant zoonotic potential. The genus *Anisakis*, for example, is known to cause anisakiasis, an infection that can occur after eating infected fish [5,31]. The genus *Gnathostoma*, which includes several species such as *Gnathostoma spinigerum*, is responsible for infections that can result in serious complications, including gnathostomiasis in humans, often associated with the consumption of raw fish or other aquatic animals [35,36]. Cestodes, especially those from the *Diphyllobothriidae* family, are also relevant in the zoonotic context. Species such as *Diphyllobothrium latum*, known as the “fish worm”, can be acquired through the consumption of raw or undercooked fish and are responsible for intestinal infections in humans [31].

Due to the widespread use of vaccines as a preventive measure, viral zoonoses are controlled, thus reducing the impact of these diseases, which are highly contagious and can spread rapidly in fish populations and aquatic ecosystems, causing significant losses to the fishing industry. Therefore, in this review, viral diseases were not addressed prominently, probably due to the effectiveness of vaccines against existing viruses [37].

Fungi, on the other hand, are considered opportunistic pathogens and have a relatively low economic and health impact but are problematic when hosts are exposed to certain stressful conditions or have their immune defenses compromised [38].

Since zoonotic agents related to emerging aquaculture activities can pose threats to public health, significant research efforts are being made in order to determine adequate explanations behind the occurrence of such pathogens. These efforts have catalyzed a multisectoral and interdisciplinary movement in support of One Health approaches, which recognize the interconnectedness of promoting the health of people, animals, plants, and the environment [5,6,39].

Some ways in which the One Health concept can be applied are through the development of surveillance systems that monitor fish health in conjunction with human and environmental health [40]. This also includes the development of epidemiological studies that examine the prevalence, distribution and risk factors of zoonotic diseases transmitted by fish [5], as well as assessments of the quality of water and aquatic ecosystems [41].

To be successful, the One Health approach needs to be applied through collaborative strategies [42]. Global challenges require international collaboration in order to be tackled more efficiently and in an integrated manner. Interdisciplinarity is emerging as a collaborative strategy that is constantly growing in society and in production assessment bodies such as the Coordination for the Improvement of Higher Education Personnel—CAPES. This growth can be justified by the transversal nature of interdisciplinarity, which extends beyond disciplinary boundaries, articulating, transposing and generating concepts, theories and methods, thus transcending the limits of disciplinary knowledge. It is distinguished by the establishment of connections between different levels of reality, logic and ways of co-producing knowledge [43,44].

However, there are many challenges to applying interdisciplinarity, such as institutional and communication barriers, resource constraints within universities and research bodies, different priorities and incentives within each discipline, establishing data-sharing protocols, as well as requiring more time and effort to plan, implement and evaluate due to the possibility of co-creation that exists in interdisciplinary groups.

The aim of this study was to carry out a systematic literature review to analyze and synthesize the existing evidence on zoonotic agents in fish, approaching the subject from the perspective of the interdisciplinary One Health concept. This research seeks to fill a specific gap in the scientific literature by exploring how interdisciplinarity has been applied in the investigation of aquatic zoonoses, with a focus on cooperation between different areas of knowledge. To this end, the study examines, from the sample of selected articles, the presence of elements that indicate the integration of multiple disciplines in scientific production and communication on zoonotic agents in fish from the perspective of One Health.

## 2. Materials and Methods

This systematic literature review was conducted according to the Preferred Reporting Items for Systematic Reviews and Meta-Analyse (PRISMA) guidelines [45].

### 2.1. Review Protocol and Guiding Question

In order to carry out this review, a review protocol was drawn up beforehand, which was tested and refined to ensure the correct procedure for searching, extracting, analyzing and transferring data, using a highly sensitive search strategy to respond to the proposed objective. In order to guarantee sensitive descriptors to search for suitable studies in a non-randomized manner, a search was carried out with DeCS/MeSH terms using controlled descriptors.

The guiding question for this research was defined based on the PICOS mnemonic strategy, where P (population to be studied) was production fish—freshwater or marine; I (intervention) was bacterial, parasitic or viral diseases; C (comparison) was healthy fish; O (outcome) was the concept and application of the term “One Health”; and S (study type) allowed only experimental studies.

### 2.2. Eligibility Criteria

The documents that were eligible for the final sample were full-text research articles available in the databases, well-designed experimental studies and studies in any language, with no time limit applied. Duplicate articles were considered only once. Documents retrieved in the form of editorials, letters to the editor, abstracts, expert opinions, other reviews, correspondences, reviews, book chapters, theses and dissertations, abstracts, lectures and books or book chapters were excluded.

A study was considered relevant when (1) it dealt with primary research published in research article format, (2) it included an evaluation of zoonotic agents in production fish, whether freshwater or marine (ornamental fish were removed from the sample), and (3) it evaluated and discussed zoonotic agents in production fish based on the One Health concept and its application.

### 2.3. Sources of Information

The studies were systematically identified by a high-sensitivity electronic search in the databases SCOPUS (Elsevier), Science Direct (Elsevier) and PubMed Central: PMC until 18 December 2023. The CAPES Periodicals Portal was used to access the databases through the proxy of the Federal University of Grande Dourados (UFGD, Dourados, MS, Brazil).

### 2.4. Search Strategy and Selection of Studies

For the peer review of the databases, a search strategy was used using the controlled descriptors DeCS/MeSH and Boolean operators in a single intersection (“Fisheries” OR “Fish” OR “Fishes”) AND (“Fish diseases” OR “Bacterial infections” OR “Virus diseases” OR “Viral zoonoses” OR “Parasites”) AND (“One health”). The search was carried out equally in all the databases based on the PICOS strategy adopted.

The screening stage was carried out by peers who read the titles, abstracts and keywords of the articles found. The full texts of the relevant articles were examined and selected according to the eligibility criteria. Microsoft Excel^®®^ 2008 software was used during all screening stages.

### 2.5. Data Collection Process

A data extraction form was drawn up specifically for the purposes of this study, including information on the identification of the publication (title of the article, DOI, authors, affiliation of the authors, institution, country of origin, language and year of publication), information on the scientific journal/journal (name and scope/objective), methodological aspects of the study (description of the experiment carried out, location of the study, variables analyzed and results found), data on the zoonosis (classification of the zoonotic agent—virus, bacterium or parasite and its respective species) and data concerning the fish (type of water—fresh or marine, species of fish). The limitations and conclusions of the studies were analyzed. All the variables obtained after the data collection process were tabulated using Microsoft Excel^®®^ 2008 software.

### 2.6. Assessment of Methodological Quality and Risk of Bias

A critical assessment of methodological quality was carried out using the CASP 2018 instrument for experimental studies (CASP Randomized Controlled Trial Standard Checklist). The risk of bias was considered low in all the studies included in the final sample of this review. The levels of evidence of the articles that constituted the final sample were classified using the Joanna Briggs Institute (JBI) method, where 1B-randomized trials had narrow confidence intervals. All the studies selected had a high level of evidence because they were individual studies with experimental designs.

### 2.7. Interdisciplinarity

The authors and co-authors were counted within each article and each journal, and their respective self-declared affiliations in the articles were subdivided according to the CAPES 2022 Knowledge Area (Basic Area—2nd level), available at: https://www.gov.br/capes/pt-br/acesso-a-informacao/acoes-e-programas/avaliacao/instrumentos/documentos-de-apoio/tabela-de-areas-de-conhecimento-avaliacao, accessed on 28 March 2024. Thus, authors who self-declared in their affiliations that they belonged to a veterinary medicine institution or department were classified as belonging to the CAPES Knowledge Area—Veterinary Medicine.

The information on the scope of each journal linked to at least one scientific article that constituted the final sample was collected directly from the journal’s website. On the Sucupira Platform—CAPES (https://sucupira.capes.gov.br/, accessed on 28 March 2024), the Qualis Reference methodology used in the 2017–2020 Quadrennium was used. With this classification, the journals were classified according to the areas with publications in a four-year period, corresponding to the journals that had at least one publication in the evaluation area, and the parent area, which was the journal that had the highest number of publications in the evaluation area.

In the 2017–2020 quadrennium, 28,417 journals were evaluated, which were distributed in 49 CAPES evaluation areas. Of these, 1087 journals are from the “interdisciplinary” parent area (3.8%), 371 journals from the “veterinary medicine” parent area (1.3%), 180 journals from the “animal science/fishery resources” parent area (0.6%) and 1187 journals from the “biological sciences I, II and III” parent areas (4.2%).

In this way, it was possible to list and quantify elements that served to explore signs of interdisciplinarity in scientific production that were also in the journals in which this production was reported. The graphs and spreadsheets were generated using Microsoft Excel^®®^ and Minitab 17 Statistical Software LLC (2021).

## 3. Results

**Figure 1:**
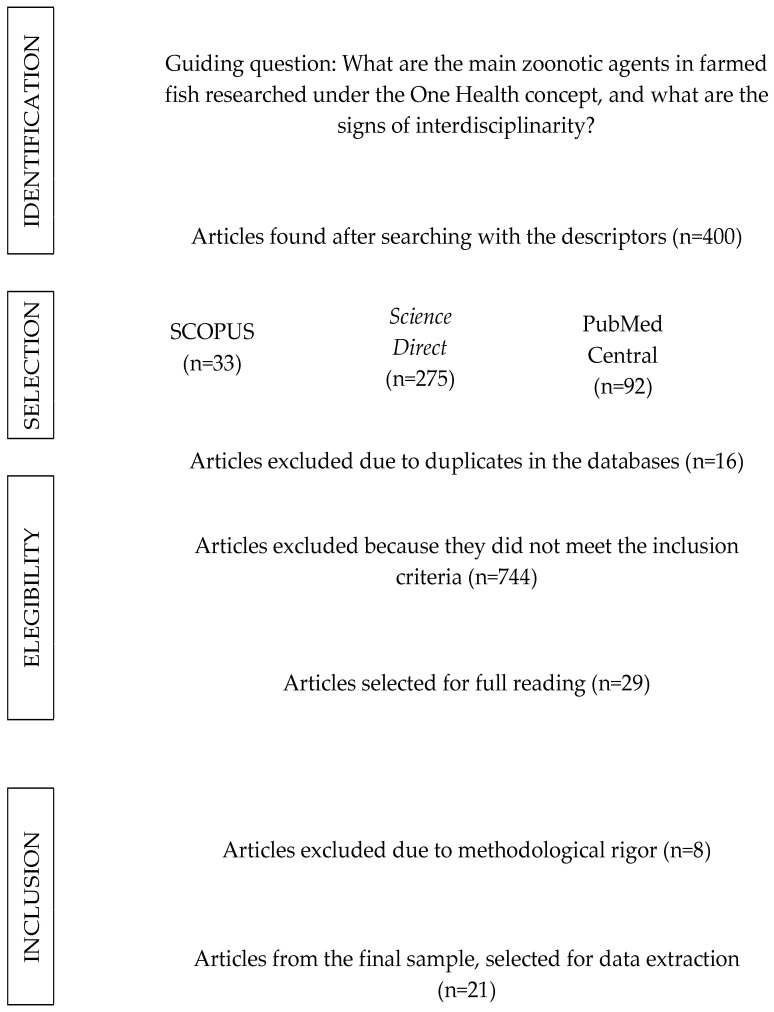
Flowchart of the article selection process for the final sample in the systematic review. Dourados/MS, Bazil, 2024.

A total of 918 documents were identified, 400 of which were classified as research articles. Of the total, 29 were identified as potential sources of data of interest, and of these, 21 publications were considered eligible and were included for data extraction due to their methodological robustness (Figure 1).

Some of the reasons why most of the articles were discarded at the screening stage were (1) the fish was cited in the article as an intermediate host, but the animal used as the experimental object was a mammal, mollusk, crustacean, bird or other animal; (2) the article was a review, report, protocol or book chapter; (3) the article carried out surveys and/or questionnaires with companies or rural communities on the subject; (4) the fish analyzed were ornamental fish; and (5) there were inconsistencies, errors or a lack of essential methodological information, such as sample numbers, animal species or diseases analyzed, among others.

### 3.1. Publication Identification

Articles published from 2018 to 2024 were retrieved, with 85.7% published in the last 5 years and all in English. The 21 articles were carried out in 13 different regions of the world, including Australia (2), Brazil (4), China (1), South Korea (1), United States (3), Mediterranean Sea (2), Italy (2), Japan (1), Malaysia (1), Peru (1), United Kingdom (1), Turkey (1) and Vietnam (1).

Among the publications, the regions with the highest incidence of parasitic and bacterial zoonoses in fish were Brazil and the United States. Australia, South Korea, the Mediterranean Sea, Italy, Japan, Turkey and Vietnam had parasitic zoonoses in fish. Bacterial zoonoses were found in China, Malaysia, Peru and the United Kingdom.

### 3.2. Methodological Aspects

The articles retrieved evaluated parasitic (66.7%) or bacterial (33.3%) zoonotic agents. No articles evaluating viruses in fish were found for the descriptors selected. In total, 7129 fish of different species were sampled. In total, 61.9% of the fish found were from freshwater environments and 38.1% from marine waters. None of the selected articles evaluated freshwater and marine fish at the same time.

The parasites affecting freshwater fish were *Posthodiplostomum minimum*, *Enterocytozoon schreckii*, *Eustrongylides excisus*, *Rohdella amazonica* n. spp., *Apatemon hypseleotris*, *Clinostomum* spp., *Parvitaenia* spp. and *Camallanus* spp.

The parasites that affected marine fish were *Trypanorhyncha* spp., *Henneguya archosargus*, *Cuculanus genypteri*, *Cuculanus pulcherrimus*, *Dichelyne sciaenidicola*, *Procamallanus halitrophus*, *Anisakis simplex*, *Sparicotyle chrysophrii*, *Kudoa hexapunctata*, *Anisakis* spp., *Contracaecum* spp., *Hysterothylacium* spp. and *Toxoplasma gondii*. The freshwater and marine fish species infected by parasites are described in Table 1.

The bacteria that affected freshwater fish were *Vagococcus salmoninarum*, *Aeromonas* spp., *Aeromonas hydrophila*, *Klebsiella pneumoniae* and *Yersinia ruckeri*. The fish species found in the sample articles that were among the most susceptible to bacterial diseases were Tilapia (*Oreochromis* spp.) and Trout (*Oncorhynchus* spp.). No species of bacteria that affect marine fish and that have zoonotic potential were found (Table 2).

**Figure 2:**
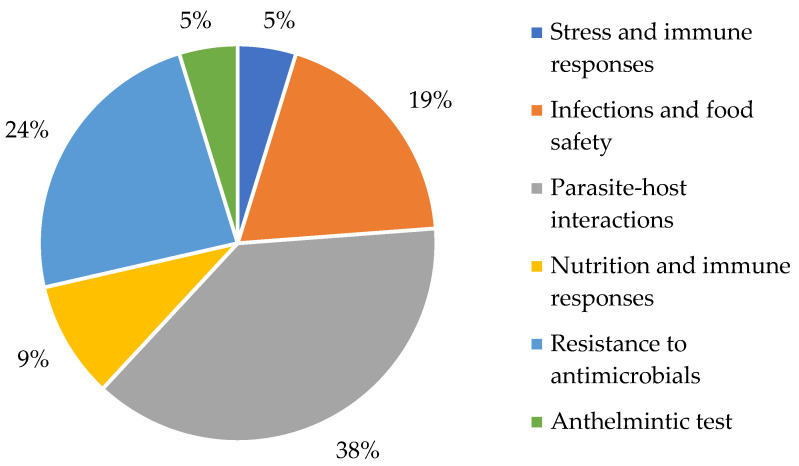
One Health approaches used in the 23 articles that constituted the sample for the systematic review.

The One Health approaches used in the 21 articles that constituted the sample were divided according to their objectives and rationales discussed in the body of the text into parasite–host interactions (38%; [46,47,48,49,50,51,52,53]), antimicrobial resistance (24%; [8,54,55,56,57]), infections and food safety (19%; [9,58,59,60]), nutrition and immune responses (9%; [61,62]), stress and immune responses (4%; [10]) and anthelmintic testing (4%; [63]) (Figure 2).

### 3.3. Institutionality, Affiliation and Interdisciplinarity

The articles found were published in 16 journals, totaling 131 authors from 60 institutions (Table 3).

**Figure 3:**
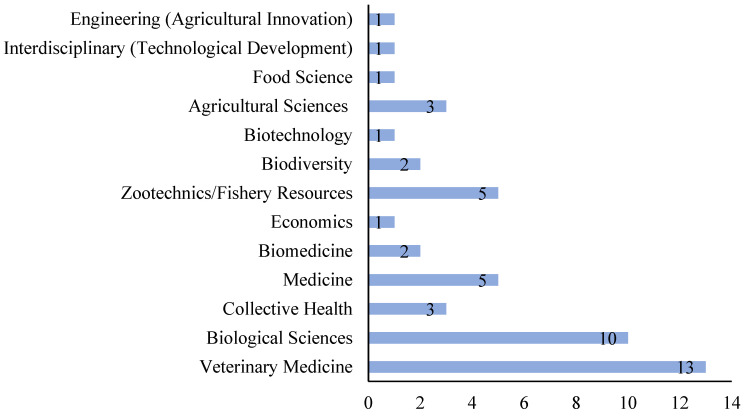
Self-declared areas of affiliation of the authors and co-authors in 23 articles, classified according to the CAPES Areas of Knowledge.

The areas of activity self-declared by the authors, shown in the footnotes of the authorships contained in the 21 articles, were based on the institutions (department or faculty) revealed in the affiliations and classified according to the CAPES Area of Knowledge. The areas of affiliation of the authors that had the highest counts were veterinary medicine (13), biological sciences (10) and zootechnics/fishery resources (5) (Figure 3).

**Figure 4:**
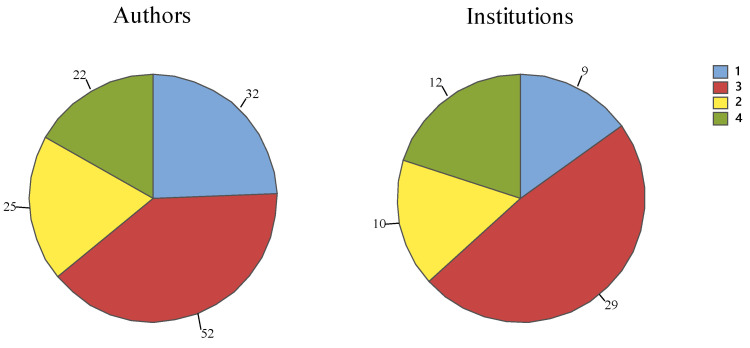
The number (frequency) of authors and the number of institutions in the articles in relation to the number of CAPES Areas of Knowledge (1, 2, 3 or 4 different areas) self-declared in the affiliation of the systematic review articles (https://www.gov.br/capes/pt-br/acesso-a-informacao/acoes-e-programas/avaliacao/instrumentos/documentos-de-apoio/tabela-de-areas-de-conhecimento-avaliacao, accessed on 28 March 2024).

Among the main areas of knowledge self-declared by the authors in the affiliations, a pattern was observed. The authors and institutions of the articles are generally divided into the following three areas (Figure 4): Veterinary medicine, biological sciences and zootechnics/fishery resources or medicine (Figure 3).

**Figure 5:**
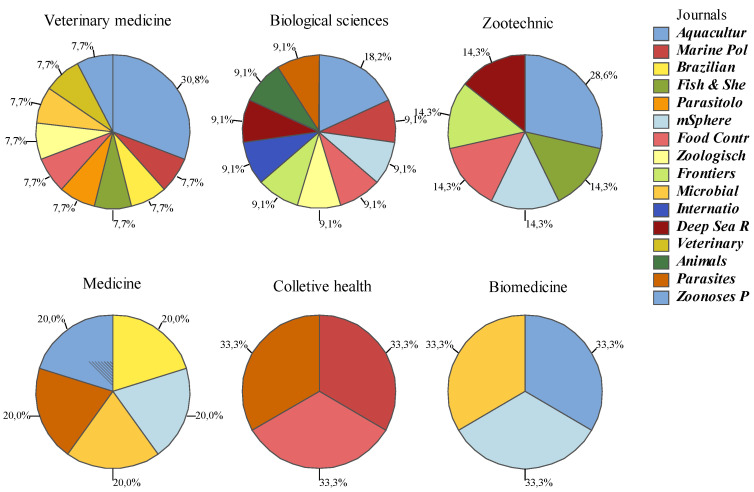
Main areas of knowledge according to the CAPES classification self-declared by the authors in the affiliations and journals in which their manuscripts were published.

The journals Parasitology Research and Veterinary World had only articles published in the area of veterinary medicine, International Journal of Food Microbiology and Animals had only articles published in the area of biological sciences, Frontiers in Marine Science and Deep Sea Research Part I: Oceanographic Research Papers had articles published in the areas of animal science/fishery resources and biological sciences, and Brazilian Journal of Veterinary Medicine and Zoonoses and Public Health had articles published in the areas of medicine and veterinary medicine (Figure 5).

Based on these areas of knowledge, it was observed that 39.7% of the authors of the articles in the sample are professionals from three areas of knowledge, with 19.1% from two areas of knowledge, 24.4% from the same area of knowledge and 16.8% from four areas of knowledge. The same pattern was evident for the institutions.

Most of the institutions self-declared by the authors have researchers from three areas of knowledge (48.3%), 16.7% from two areas of knowledge and 20.0% from four areas of knowledge, while 15.0% of the affiliations have only one area of knowledge, probably because all the authors and co-authors are from the same institution, department and/or academic background (Figure 4).

*Aquaculture*, *Marine Pollution Bulletin*, *mSphere*, *Food Control*, *Microbial Pathogenesis and Parasites & Vectors* were the journals that had three or more areas of knowledge in their publications (Figure 5).

The journals *Aquaculture*, *Frontiers in Marine Science*, *Animals* and *Zoonoses and Public Health* had the term “Interdisciplinary” or “Multidisciplinary” in the scope or objective of the journal (see website). In the CAPES classification, when the areas with publications in the four-year period were evaluated, only the journals *Aquaculture* and *Deep Sea Research Part I: Oceanographic Research Papers* did not have publications classified as “Interdisciplinary” (Table 4).

The 16 journals in the sample are classified as being in the areas of veterinary medicine (4/16), biodiversity (4/16), biological sciences (3/16), animal science/fishery resources (3/16) and food science (2/16) by CAPES’ parent area (Table 4).

## 4. Discussion

### 4.1. Publication ID

The concept of One Health is not new and can be traced back at least 200 years [64]; however, the evolution of the term to One Health occurred in the 21st century [65]. This explains the fact that the articles found are recent and highlights the novelty of research linking the risk of transmissible zoonotic diseases in fish with the One Health concept.

Asian, European and American countries were the source of most of the papers found, possibly because these countries are the source of ∼92% of total fishery production [66], and consequently, where there is greater production, there is greater opportunity to research these diseases and infections.

### 4.2. Methodological Issues

Much is known about fish diseases, their prevention and/or control [5,67]; however, the general perception is that there are few zoonotic diseases considered important in the aquatic environment compared to other environments and animal production species [68]. While this may be correct, there is a possibility that this is an underestimate due to a lack of awareness, monitoring, surveillance and/or definitive diagnosis rather than the absence and importance of these diseases [69].

Most aquatic production systems face problems related to parasitic infections, which challenge fish health and welfare, as well as serving as a gateway for secondary pathogens. Controlling parasitic infections in fish is key to preventing their impact on human health and wild fish populations [50]. Fish with high parasite infestation have low immunity and delayed growth [70] and are consequently more likely to contract viral and/or bacterial infections.

Several species of parasites have been identified as potential agents of infection in humans, especially when fish are consumed raw or undercooked. Of the parasites found in this review, we encountered *Clinostomum* spp., which is a genus of trematodes that can cause infections in humans, as demonstrated in a case of laryngopharyngitis resulting from the ingestion of contaminated fish [71], *Kudoa hexapunctata*, which has been associated with foodborne illness in humans [72,73], *Cucullanus* spp., specifically *Cucullanus genypteri* [74], *Anisakis simplex*, which causes allergic reactions and gastrointestinal infections [75], and *Toxoplasma gondii*, which, although not a parasite exclusively associated with fish, can be transmitted through the consumption of contaminated meat, including fish. Infection with *T. gondii* can result in toxoplasmosis, a disease that can be serious, especially in immunocompromised individuals. Other parasites mentioned, such as *Henneguya archosargus* and *Eustrongylides excisus*, have been identified in fish, but documentation of direct human infections is scarce. However, the possibility of infection should not be ruled out, especially in contexts where the consumption of contaminated fish is common.

The classic form of parasite control is the use of antiparasitic drugs, but the impressive ability of parasites to adapt to environmental changes and aspects linked to natural or directional selection challenge effective and long-lasting parasite control [76]. In addition, different legislation on the use of drugs in different countries can hinder prevention and control measures [77].

Bacteria are also a major concern in fish farming due to the high mortality they cause and the consequent emergence of antibiotic-resistant bacterial pathogens [8]. Among the bacteria found in the sample articles, the genus *Vagococcus* is gram-positive, and the others are gram-negative bacteria (*Aeromonas*, *Klebsiella* and *Yersinia*). The higher incidence of gram-negative bacteria may be related to the composition of the cell wall, which has an outer membrane composed mainly of lipopolysaccharides. Lipopolysaccharides are endotoxins that tend to trigger severe inflammatory responses, as well as reducing the sensitivity and effectiveness of antibiotics [78].

Although *V. salmoninarum* is primarily an aquatic pathogen, there is limited evidence of its potential to cause human infection. References do not directly report any cases of human infection associated with this bacterium [79,80]. However, some studies have observed that certain *Vagococcus* species, such as *V. fluvialis*, have been isolated from human clinical samples, including blood, peritoneal fluid and wounds [81,82]. This suggests that *Vagococcus* species, including *V. salmoninarum*, may have the potential to infect humans opportunistically, particularly those with compromised immune systems or those who have had direct contact with contaminated aquatic environments [80].

Other authors have also documented cases of infection in humans by various bacteria found in this review, such as *Aeromonas* spp. and *Aeromonas hydrophila*, which are recognized as pathogenic in humans, causing a variety of infections, including gastroenteritis, wound infections and septicemia, especially in immunocompromised individuals. *Aeromonas hydrophila*, in particular, has been associated with infections in humans, with studies showing that the bacterium can be isolated from clinical samples such as feces and blood [83]. *Klebsiella pneumoniae* is a well-documented human pathogen responsible for a wide range of infections, including pneumonia, urinary tract infections and bacteremia, a common cause of hospital-acquired infection, with significant prevalence in intensive care units [84,85]. *Yersinia ruckeri*, although primarily a fish pathogen, has been reported in rare cases of human infection. The first documented case of infection in humans was in a patient with a wound, indicating that, although rare, *Y. ruckeri* can act as a zoonotic agent [86].

Effective vaccination strategies and standardization of protocols for many common bacterial infections in fish farms are still lacking, and antibiotics tend to be the first choice to treat them [87]. However, the overuse of antibiotics and/or the use of unauthorized antibiotics can promote antimicrobial resistance, compromising the effectiveness of infection treatments and human health [8].

Because they are considered opportunistic pathogens, i.e., they cause infections in the presence of environmental stress, injury, sudden changes in temperature or other conditions that compromise the fish immune system, the most widely used methods for control are proper management and nutrition, which, in addition to improving fish performance, improve immune status and contribute to greater resistance and resilience to bacterial infection [4,61,87].

No articles on viral zoonotic diseases were found with the descriptors selected for literature sampling. Some of the possible reasons for this lie in the fact that mass vaccination for viruses is a common practice in aquaculture. In many countries, the restriction of entry and exit from facilities, disinfection and sterilization are also common practices in aquaculture, mainly due to the existing strictness of legislation for viral diseases [88,89]. Another explanation may be related to the increased transmission of viruses (hepatitis A, E, norovirus and rotavirus) by contaminated seafood, such as shrimp and shellfish [90], which were not the subject of this review.

The One Health approaches in the selected articles were based on global concerns about existing parasite–host interactions, antimicrobial resistance, nutrition and immune responses and the relationship of infections with food safety (Figure 2), which corroborates so many other studies that have shed light on the subject of zoonotic diseases in fish [5]. These emerging themes require special attention to the role of interdisciplinary approaches in providing knowledge, more sustainable experiences and a better understanding of the importance of One Health policies [91].

### 4.3. Institutionality, Affiliation and Interdisciplinarity

Two of the principles of the One Health approach are interdisciplinarity and multi-sector collaboration [65]. It is known that interdisciplinary thinking is best learned in truly diverse groups of students, including science, technology, engineering, mathematics and medicine, as well as humanities, arts and social sciences [92]. Therefore, these areas need to be present, interacting and partnering through different research groups or faculties within a university. The primary difficulty in implementing the unified One Health approach in these cases may lie in the major political, legal, ethical, financial, capacity and social barriers and complexities [93].

Possibly, the sample of published articles portrays a professional class bias, where interdisciplinarity seems too discreet or compromised by the greater occurrence of areas such as veterinary medicine, biological sciences and zootechnics/fishery resources. These results corroborate [65], where current health problems are reportedly complex and often approached from a purely medical, veterinary or ecological point of view and are therefore unlikely to engender sustainable mitigation strategies.

For interdisciplinary research to advance, it is necessary to explore beyond issues involving disciplines traditionally linked to medicine or agricultural sciences, such as genetics, animal nutrition and experimentation and pathogen–host relations, among others. The reflection that this provokes is not to diminish the significance of these important areas of scientific activity but to question the low frequency of professionals allocated to faculties or departments in the areas of economics, engineering, food science and interdisciplinary/technological development (1 in each case (Figure 3)). It is plausible to argue about the importance of socio-economic studies, market studies, the pharmaceutical industry, technical assistance, labor, the challenges of technification, professional profiles, behavioral issues of producers, literacy or illiteracy and cooperative culture, among others, in high-level studies that cover the production and zoonoses of farmed fish from an interdisciplinary perspective.

Perhaps classism, in this case, can be understood as “structures” or “clusters” of areas of knowledge, institutions or faculties, which, in a more consolidated way, restricts greater signs of interdisciplinary cooperation and communication. This could have an impact on new discoveries and changes towards greater sustainability. It seems that the sample retrieved from this systematic review represents a relatively select group of certain professions and areas of knowledge. Perhaps, this is an indication of the difficulty of operationalizing high-level research with broad concepts that require a more interdisciplinary contribution, such as One Health.

A pattern was observed in which, on the one hand, journals directly related to zoonotic diseases refer to medical, veterinary or human professionals. On the other hand, journals associated with parasites, which use a derivation of this term in their titles, are associated with collective health, medicine and biology. On the other hand, journals about animals, such as fish, referred to authors working in zootechnics, biology and veterinary science.

It is plausible to assume that there are factor(s) restricting professionals from other areas from recognizing, identifying, collaborating and even becoming authors in these journals or participating in the research teams, institutes or faculties that prevailed from this systematic review.

*Aquaculture* was the journal with the highest frequency of articles in the sample (6/23; 26.08%). It also had the highest number of authors and institutions involved (Table 3). The term “Interdisciplinary” appears in the scope definition of this journal on the journal’s website, and it does not have an “Interdisciplinary” classification in the criteria adopted by CAPES, as revealed on the Sucupira platform (Table 4).

Secondly, with regard to the frequency of articles from the same journal in the sample of 16 different journals, *Food Control* and *Microbiol Pathogens* had two counts (2/23; 8.89% each). The term “Interdisciplinary” is not relevant in the scope of these journals, as they are classified by CAPES as journals in the parent areas of food science and biological sciences (Table 4).

Of the 23 journals, none (0%) were registered as being in the “Interdisciplinary” parent area by CAPES (Table 4). Most of the journals (14/16; 87.5%) had articles published at least once in the parent area of interdisciplinarity in the four-year period analyzed, with the exception of *Aquaculture* and *Deep Sea Research Part I: Oceanographic Research Papers* (Table 4).

Thus, the journal Aquaculture, which had the highest frequency among the articles selected (6/23), did not have any articles published that corresponded to the “Interdisciplinary” area in the CAPES classification. However, it is worth noting that the absence of a publication linked to the area of interdisciplinarity does not imply that there is not a wide range of areas in which journal articles are classified, according to CAPES.

The journal *Deep Sea Research Part I: Oceanographic Research Papers*, for example, had publications in the areas of engineering, mathematics/probability and statistics and geosciences, among others, but no article from this journal in the four-year period analyzed was classified as “Interdisciplinary”. The same was observed for the journal *Aquaculture*, which was restricted to publications allocated according to the CAPES criteria in biodiversity, geosciences and veterinary medicine (Table 4). Directions and scientific contributions in the One Health area could be discussed or adjusted with these statistics, presented here, as an impetus to leverage and strengthen One Health as a backdrop for the sustainable development of the fish/fishery chain and other agricultural commodities. Thus, One Health would be an approach that encompasses, approximates and dilutes distances between the pillars that formed the primordial concept of sustainable development.

The sample of articles and journals in this study perhaps portrays a collection that refers to a background, or portrays a means of executing science. In this respect, the success and achievement of publication in high-level journals (all of which are “A” in the criteria of the Sucupira Platform—CAPES) is traditionally justified by the search for depth on a given point. In contrast, interdisciplinary work is perhaps better characterized as attempts to overlap knowledge, with complementarities, to create something new, which also expands on the surface. Perhaps, this is a difficulty deliberately faced by the current generation of researchers affected by One Health in their day-to-day work.

Alternatively, researchers may be more motivated or trained to deepen their knowledge in their specific areas, including collaboration between peers, usually within the same faculty, or in research teams consisting of graduates from similar undergraduate and postgraduate programs.

It is likely that the advancement of scientific production, with One Health as a backdrop, depends on the disruption of a way of engaging in and thinking about science. While reforms at the institutional and institutional governance levels are slower, this progress, in the short term, may depend on the attitudes of select scientists who sacrifice something to form collaborative arrangements that configure a more representative universe, such as greater unity between faculties, and multilateralism in favor of greater impact and innovation in society and academia.

Therefore, this systematic review also fortuitously shed light on the subject of interdisciplinarity in research associated and/or trying to connect with the One Health concept, revealing signs that interdisciplinarity may be limited. This, in turn, may have restricted new knowledge and perspectives on the subject of zoonoses in farmed fish. The ways in which researchers from different areas connect with the One Health concept, as well as the ways in which scientific communication vehicles approach or deal with the topic, could perhaps be the subject of future research. However, it is curious to note the absence of various faculties and areas of knowledge in the statistics that refer to a topic of collective interest, such as socioeconomics and finance, among others. Perhaps, revisiting and better understanding the genesis of the One Health concept in the different institutions, the different traditions and ways of cooperating and conducting research and the permeability of researchers from different areas in the various high-level journals are paths that could be taken.

In short, we argue that the results point to discreet interdisciplinarity, and that discussing and better understanding this aspect could be a key to leveraging One Health’s theoretical and practical contributions to issues involving sustainable production, food systems and food consumption, as well as a more prosperous and healthy life for humanity.

Interdisciplinary research on the subject needs to be based on a systemic vision such that multidisciplinary teams can engage in dialog and contribute scientifically to structuring/delineating research in line with the real demands of society. This requires the participation of multiple stakeholders from different sectors such as academia, industry and governments.

### 4.4. Limitations of the Study

One of the main limitations of this study is the exclusion of certain databases, which probably resulted in the omission of numerous relevant publications. This decision was made based on the standardization of the eligibility criteria established in the methodology, which aimed to guarantee the homogeneity and quality of the sources selected. Although this approach was necessary to reduce bias and guarantee the consistency of the data analyzed, it may also have restricted the scope of the review, limiting the inclusion of important works that could contribute to a more complete and diverse analysis of the topic. In addition, few studies relate fish diseases to the concept of One Health. The filtering of articles using the descriptor “One Health” meant that many articles on fish diseases were not included in the final sample.

The lack of global standardization of descriptors in the area of agricultural sciences is also a major limitation when searching, as it limits the reader at the time of the search.

## 5. Conclusions

The One Health approaches identified in the articles reviewed reflect a growing global awareness of critical issues such as parasite–host interactions, antimicrobial resistance, nutrition, immunity and food safety. These topics inherently require integrated and collaborative responses, underscoring the importance of interdisciplinary efforts to advance scientific understanding and formulate sustainable public health policies.

Despite this recognition, the results reveal a concentration of research in isolated fields—mainly veterinary medicine and biological sciences—with limited interdisciplinary interaction. This lack of integration can hinder the development of more comprehensive and effective strategies for tackling zoonotic diseases in fish. A truly robust application of the One Health concept requires active collaboration between various disciplines, including environmental sciences, public health, epidemiology and social sciences. This synergy enhances the ability to generate holistic insights, promote innovation and implement more resilient and sustainable solutions to complex health challenges.

## Figures and Tables

**Table 1:** Final sample of articles included in the systematic review that researched parasitic zoonotic agents in fish.

Authors	Location	Zoonotic Agents	Water	Fish Species
Bader et al. [46]	USA *	*Posthodiplostomum minimum*	Fresh	*Lepomis macrochirus*
Couch et al. [10]	USA *	*Enterocytozoon schreckii*	Fresh	*Oncorhynchus tshawytscha*
Castiglione et al. [9]	Italy	*Eustrongylides excisus*	Fresh	*Ameiurus melas*, *Carassius auratus*, *Silurus glanis*, *Lepomis gibbosus*, *Cyprinus carpio*, *Anguilla anguilla*, *Tinca tinca*, *Micropterus salmoides*, *Atherina boyeri*, *Chelon ramada* and *Pseudorasbora parva*
Hayes et al. [47]	United Kingdom	*Cryptosporidium* spp.	Fresh	*Salmo salar*, *Salmo truttan* and *Cottus gobio*
Swiderski et al. [48]	Brazil	*Rohdella amazonica*	Fresh	*Colomesus psittacus*
Shamsi et al. [49]	Australia	*Apatemon hypseleotris*, *Clinostomum* spp., *Parvitaenia* and *Camallanus*	Fresh	*Retropinna semoni* and *Hypseleotris* spp.
Palomba et al. [50]	Mediterranean Sea **	*Trypanorhyncha* spp.	Marine	*Lepidopus caudatus*
Casal et al. [51]	Brazil	*Henneguya archosargus*	Marine	*Archosargus probatocephalus*
Di Azevedo et al. [52]	Brazil	*Cucculanus genypteri*, *Cucculanus pulcherrimus*, *Dichelyne sciaenidicola* and *Procamallanus halitrophus*	Marine	*Genypterus brasiliensis, Micropogonias furnieri* and *Mullus argentinae*
Kumagai and Nishino [53]	Japan	*Anisakis simplex*	Marine	*Coryphaenoides acrolepis*, *Coelorhynchus japonicus* and *Coelorhynchus macrochir*
Riera-Ferrer et al. [54]	Mediterranean Sea	*Sparicotyle chrysophrii*	Marine	*Sparus aurata*
Kang et al. [55]	South Korea	*Kudoa hexapunctata*	Marine	*Thunnus orientalis*
Suthar et al. [56]	Australia	*Anisakis* spp., *Contracaecum* spp. and *Hysterothylacium* spp.	Marine	*Platycephalus richardsoni*, *Scomber australasicus*, *Pagrus auratus* and *Sillago flindersi*
Marino et al. [57]	Italy	*Toxoplasma gondii*	Marine	*Esfiraena argentina*, *Arnoglossus lateral*, *Boops boops*, *Congro congro*, *Diplodus sargus*, *Engraulis encrasicolus*, *Merluccius merluccius*, *Mullus barbatus*, *Pagellus acarne*, *Pagellus erytrinus*, *Raja clavata*, *Sardina pilchardus*, *Sarpa salpa*, *Scorpaena scrofa*, *Serrano cabrilla*, *Spicara maena* and *Trachurus trachurus*

* USA: United States of America; ** Malta Island.

**Table 2:** Final sample of articles included in the systematic review that researched bacterial zoonotic agents in fish.

Authors	Location	Zoonotic Agents	Water	Fish Species
Saticioglu et al. [58]	Turkey	*Vagococcus salmoninarum*	Fresh	*Oncorhynchus mykiss*
Yamaguchi et al. [59]	Vietnam	*Aeromonas* spp.	Fresh	*Anabas testudineus*, *Channa striata*, *Clarias fuscus*, *Cynoglossidae* spp., *Cyprinus carpio*, *Mastacembelus* spp., *Mugil* spp., *Oreochromis* spp. and *Pangasius bocourti*
Fauzi et al. [8]	Malaysia	*Aeromonas* spp.	Fresh	*Oreochromis* spp., *Clarias gariepinus* and *Pangasianodon hypophthalmus*
Moya-Salazar et al. [60]	Peru	*Aeromonas hydrophila*	Fresh	*Oncorhynchus mykiss*
Cheng et al. [61]	China	*Aeromonas hydrophila*	Fresh	*Cyprinus carpio*
Vaneci-Silva et al. [62]	Brazil	*Klebsiella pneumoniae*	Fresh	*Oreochromis niloticus*
Sibinga et al. [63]	USA *	*Yersinia ruckeri*	Fresh	*Oncorhynchus mykiss*

* USA: United Stated of America.

**Table 3:** Authors, journals, the number of authors (NA) and the number of institutions (departments, faculties, research groups; NI) involved in the publications that constituted the final sample of articles.

Authors	Journal	NA	NI
Saticioglu et al. [58]; Vaneci-Silva et al. [62]; Riera-Ferrer et al. [54]; Shamsi et al. [49]	Aquaculture	25	12
Yamaguchi et al. [59]	Marine Pollution Bulletin	16	7
Moya-Salazar et al. [60]	Brazilian Journal of Veterinary Medicine	7	6
Cheng et al. [61]	Fish & Shellfish Immunology	3	2
Bader et al. [46]	Parasitology Research	5	1
Couch et al. [10]	MSphere	7	3
Castiglione et al. [9]; Palomba et al. [50]	Food Control	14	7
Swiderski et al. [48]	Zoologischer Anzeiger	5	7
Sibinga et al. [63]	Frontiers in Marine Science	5	1
Casal et al. [51]; Suthar et al. [56]	Microbial Pathogenesis	8	3
Di Azevedo et al. [52]	International Journal of Food Microbiology	2	1
Kumagai and Nishino [53]	Deep Sea Research Part I: Oceanographic Research Papers	2	2
Fauzi et al. [8]	Veterinary World	6	1
Kang et al. [55]	Animals	7	1
Hayes et al. [47]	Parasites & Vectors	8	4
Marino et al. [57]	Zoonoses and Public Health	11	2
Total		131	60

**Table 4:** Presence of interdisciplinary scope and areas of classification of the journals by CAPES.

Journal	Interdisciplinary Scope	Area(s) With Publication in the Quadrennium	Parent Area
Aquaculture	Y	Biodiversity, Geosciences and Veterinary Medicine	Zootechnics/Fishery Resources
Marine Pollution Bulletin	N	Interdisciplinary	Biodiversity
Brazilian Journal of Veterinary Medicine	N	Interdisciplinary	Veterinary Medicine
Fish & Shellfish Immunology	N	Interdisciplinary	Zootechnics/Fishery Resources
Parasitology Research	N	Interdisciplinary	Veterinary Medicine
MSphere	N	Interdisciplinary	Biological Sciences
Food Control	N	Interdisciplinary	Food Science
Zoologischer Anzeiger	N	Interdisciplinary	Biodiversity
Frontiers in Marine Science	Y	Interdisciplinary	Biodiversity
Microbial Pathogenesis	N	Interdisciplinary	Biological Sciences
International Journal of Food Microbiology	N	Interdisciplinary	Food Science
Deep Sea Research Part I: Oceanographic Research Papers	N	Environmental Sciences, Biological Sciences, Engineering, Geoscience, Mathematics/Probability and Statistics	Biodiversity
Veterinary World	N	Interdisciplinary	Veterinary Medicine
Animals	Y	Interdisciplinary	Zootechnics/Fishery Resources
Parasites & Vectors	N	Interdisciplinary	Biological Sciences
Zoonoses and Public Health	Y	Interdisciplinary	Veterinary Medicine

## Data Availability

The original contributions presented in this study are included in the article. Further inquiries can be directed to the corresponding author(s).
